# Importance of Achromatic Contrast in Short-Range Fruit Foraging of Primates

**DOI:** 10.1371/journal.pone.0003356

**Published:** 2008-10-06

**Authors:** Chihiro Hiramatsu, Amanda D. Melin, Filippo Aureli, Colleen M. Schaffner, Misha Vorobyev, Yoshifumi Matsumoto, Shoji Kawamura

**Affiliations:** 1 Department of Integrated Biosciences, Graduate School of Frontier Sciences, The University of Tokyo, Kashiwa, Japan; 2 Department of Anthropology, University of Calgary, Calgary, Alberta, Canada; 3 Research Centre in Evolutionary Anthropology and Palaeoecology, School of Natural Sciences and Psychology, Liverpool John Moores University, Liverpool, United Kingdom; 4 Department of Pyschology, University of Chester, Chester, United Kingdom; 5 Department of Optometry and Vision Science, University of Auckland, Auckland, New Zealand; University of Bristol, United Kingdom

## Abstract

Trichromatic primates have a ‘red-green’ chromatic channel in addition to luminance and ‘blue-yellow’ channels. It has been argued that the red-green channel evolved in primates as an adaptation for detecting reddish or yellowish objects, such as ripe fruits, against a background of foliage. However, foraging advantages to trichromatic primates remain unverified by behavioral observation of primates in their natural habitats. New World monkeys (platyrrhines) are an excellent model for this evaluation because of the highly polymorphic nature of their color vision due to allelic variation of the L-M opsin gene on the X chromosome. In this study we carried out field observations of a group of wild, frugivorous black-handed spider monkeys (*Ateles geoffroyi frontatus*, Gray 1842, Platyrrhini), consisting of both dichromats (*n* = 12) and trichromats (*n* = 9) in Santa Rosa National Park, Costa Rica. We determined the color vision types of individuals in this group by genotyping their L-M opsin and measured foraging efficiency of each individual for fruits located at a grasping distance. Contrary to the predicted advantage for trichromats, there was no significant difference between dichromats and trichromats in foraging efficiency and we found that the luminance contrast was the main determinant of the variation of foraging efficiency among red-green, blue-yellow and luminance contrasts. Our results suggest that luminance contrast can serve as an important cue in short-range foraging attempts despite other sensory cues that could be available. Additionally, the advantage of red-green color vision in primates may not be as salient as previously thought and needs to be evaluated in further field observations.

## Introduction

Among placental mammals, only primates have evolved unique trichromatic color vision from dichromatic ancestors [1]. This is accomplished by the presence of long wave sensitive (L), middle wave sensitive (M) and short wave sensitive (S) opsins produced separately in the cone photoreceptive cells in the retina. The L and M opsins arose *via* allelic differentiation of a single L-M opsin gene on the X chromosome in platyrrhines (New World monkeys) [2], [3], resulting in the extensive polymorphism of color vision, *i.e.* trichromacy for females heterozygous for the X-linked L-M opsin alleles and dichromacy for all males and homozygous females [4]. Exceptions have only been found in two genera: *Aotus* (owl monkeys) and *Alouatta* (howler monkeys), the former being monochromatic and nocturnal, having only an M opsin allele and no functional S opsin [5], [6], and the latter being routinely trichromatic, having the L and M opsin genes juxtaposed by gene duplication on the X chromosome [7] as in catarrhines (humans, apes and Old World monkeys). A wide variation of L-M opsin allelic composition has been found among many other species of New World monkeys, ranging from diallelic, seen typically in *Ateles* (spider monkeys) and *Lagothrix* (woolly monkeys), up to pentallelic reported for *Callicebus moloch* (dusky titi) [8]–[11]. Why can New World monkeys be so variable in color vision, while tirchromacy is almost the norm in catarrhines? Because of the wide variation of color vision both within and between species, New World monkeys are excellent subjects to study the utility of color vision in natural environment and thus to elucidate the selective advantage of being a trichromat or dichromat. Visual phenotypes can be determined non-invasively through DNA analyses of the opsin gene collected from fecal samples [12], [13].

The selective advantage of trichromacy has been suggested by many studies. Colorimetric measurements of natural scenes in forests revealed that the chromaticity of foliage falls in a very narrow range of L/(L+M) value (which provides a measure of the redness provided by the ‘red-green’ chromatic channel equipped for only trichromatic primates and subserved by the midget ganglion cells [14], [15]), but spreads widely in S/(L+M) value (which provides a measure of the blueness provided by more ancient ‘blue-yellow’ chromatic channel equipped for all mammals and subserved by the small bistratified ganglion cells [16], [17]) and also in luminance values [18], [19], leading to the argument that primate trichromacy should be advantageous for detecting targets differing from the background foliage in L/(L+M) value, such as ripe fruits, young leaves, pelage and skin [19]–[22]. This trichromat advantage is supposed to be maximized during long distance viewing because the scene would contain a larger variety of background S/(L+M) and luminance values than a closer view would. In addition, during close viewing, other sensory cues, such as odors, are available and visual cues could be less important. In contrast, trichromat advantage at close viewing distance is suggested by psychophysical studies. The human visual system shows a relatively greater sensitivity to low spatial frequencies of chromatic spatial modulation than to luminance spatial modulation [23]. In addition, a statistical analysis of spatial frequencies of natural images suggests that the spatiochromatic properties of the red-green system of human color vision may be optimized for the encoding of any reddish or yellowish objects on a background of foliage at relatively small viewing distances commensurate with a typical grasping distance [24]. Other colorimetric studies incorporating nutritional measurements of primate diets support the trichromat advantage in foraging young leaves or fruits [25]–[29]. Lastly, standardized behavioral experiments demonstrated superior ability of trichromatic to dichromatic primates in detecting reddish objects against greenish background [30]–[32].

Despite these findings, behavioral observation of wild primate populations has given a limited support for trichromat advantage. In a study of wild mixed-species troops of saddleback (*Saguinus fuscicollis*) and mustached (*S. mystax*) tamarins, trichromats are further from their neighbors than their dichromatic conspecifics are during vigilance, which is explained through the potentially better perception of predation risk in trichromats [33]. Results of many other field observations are equivocal or opposite to the pattern expected of the trichromat advantage hypothesis. The study of the mixed-species troops of tamarins showed that neither the color-vision types (dichromatic or trichromatic) nor the sex of individuals had a consistent effect on the leadership of the troops to feeding trees [34]. Another study of tamarins (*S. imperator imperator* and *S. fuscicollis weddelli*) found no significant difference between females (thought to consist of trichromats and dichromats) and males (all dichromats) in their ability to locate or discriminate feeding sites [35]. No significant difference between trichromats and dichromats was found in feeding and energy intake rates in a population of capuchin monkeys (*Cebus capucinus*) [36] or in foraging time spent on different food types in another population of the same capuchin species [37]. Some modeling studies have found that many fruits eaten by spider monkeys (*Ateles geoffroyi*) or squirrel monkeys (*Saimiri sciureus*) are similarly discernible or similarly indiscernible from background foliage for both trichromats and dichromats [38]–[40]. Even a disadvantage of trichromacy has been suggested by behavioral experiments using capuchins (*Cebus apella*) and marmosets (*Callithrix geoffroyi*) for detecting color-camouflaged objects [41], [42] and a field study of capuchin monkeys (*C. capucinus*) has demonstrated a dichromat advantage in foraging for surface-dwelling insects [43].

In the present study, we investigated a free-ranging social group of black-handed spider monkeys (*Ateles geoffroyi frontatus*, Gray 1842), living in Santa Rosa National Park, Costa Rica. These monkeys have been individually identified and habituated by researchers during long-term socioecological studies [44], [45]. We focused on fruit foraging because fruits are a major component of most primate diets and fruit detection has been a classic source of debate since 19th century concerning the evolution of trichromatic color vision in primates [46]–[49]. Spider monkeys are highly frugivorous, spending 80–90% of their foraging time feeding on fruit [50], [51] and are therefore ideal for testing the importance of trichromacy *via* the red-green chromatic channel in fruit detection.

To acquire the data necessary to address our research questions, we measured the absorption spectra of the L-M opsin (visual pigment) alleles of the spider monkeys reconstituted *in vitro*. We also measured the reflectance spectra of their dietary fruit and background leaves at the field site. During behavioral data collection, which took place over eight months in 2004–2005, we focused on foraging behaviors occurring in a fruiting tree, that is, under a foraging situation where monkeys are within close proximity to the fruits. We examined whether there were any differences in foraging efficiency between dichromatic and trichromatic individuals and whether the strength of chromatic and achromatic contrasts between fruits and background leaves were significantly correlated with the monkeys' foraging efficiencies to determine which component(s) of the potential visual cues (among red-green, blue-yellow and luminance contrasts) had the greatest influence on foraging efficiency.

## Results

### Color vision phenotypes

**Figure 1:**
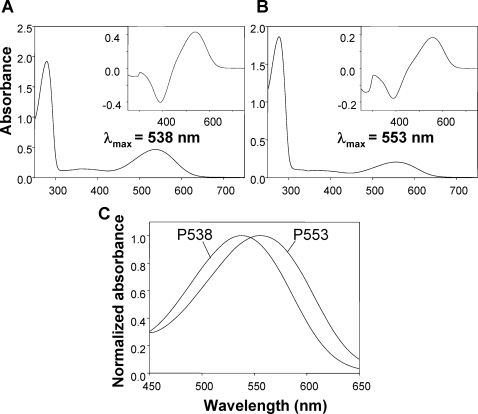
Absorption spectra of P538 (A) and P553 (B) alleles of spider monkey L-M opsin photopigments reconstituted *in vitro*. Insets: dark-light difference absorption spectra, showing that the reconstituted pigments are photosensitive. λ_max_ values are directly taken from the dark absorption spectra. (C) Normalized absorbance of the two pigments presented together, peak height being adjusted to 1.

We present the color vision phenotypes of our study subjects in Table 1. We determined these non-invasively by examining the L-M opsin gene *via* PCR amplification from fecal DNA and then by the functional reconstitution of the photopigments. Two spectrally distinct alleles were identified, one with the peak absorption maxima (λ_max_) at 553±0.7 nm (designated P553) and the other with λ_max_ at 538±0.3 nm (designated P538; Figure 1). The two alleles made two dichromat phenotypes and one trichromat phenotype possible in the population.

**Table 1:** Number of color vision phenotypes in the spider monkeys analyzed in this study

Color vision type	Female		Male		Total
	Adult/Sub-adult	Juvenile	Adult/Sub-adult	Juvenile	
Dichromat					
P538	1	1	0	1	3
P553	6	0	3	0	9
Trichromat					
P538/P553	8	1	-	-	9
Total	15	2	3	1	21

In our study group, there was no sequence variation at the amino acid level throughout the protein-coding region in each allele. When filtering effects of lens and macular pigment were taken into consideration (see Materials and Methods), the effective λ_max_ values of the P553 and P538 at the cornea were estimated to be 560 and 545 nm, respectively. These values are consistent with those given by a previous electroretinogram (ERG) flicker photometry at ∼562 and ∼550 nm for spider monkeys [8].

### Chromaticity of fruits

**Figure 2:**
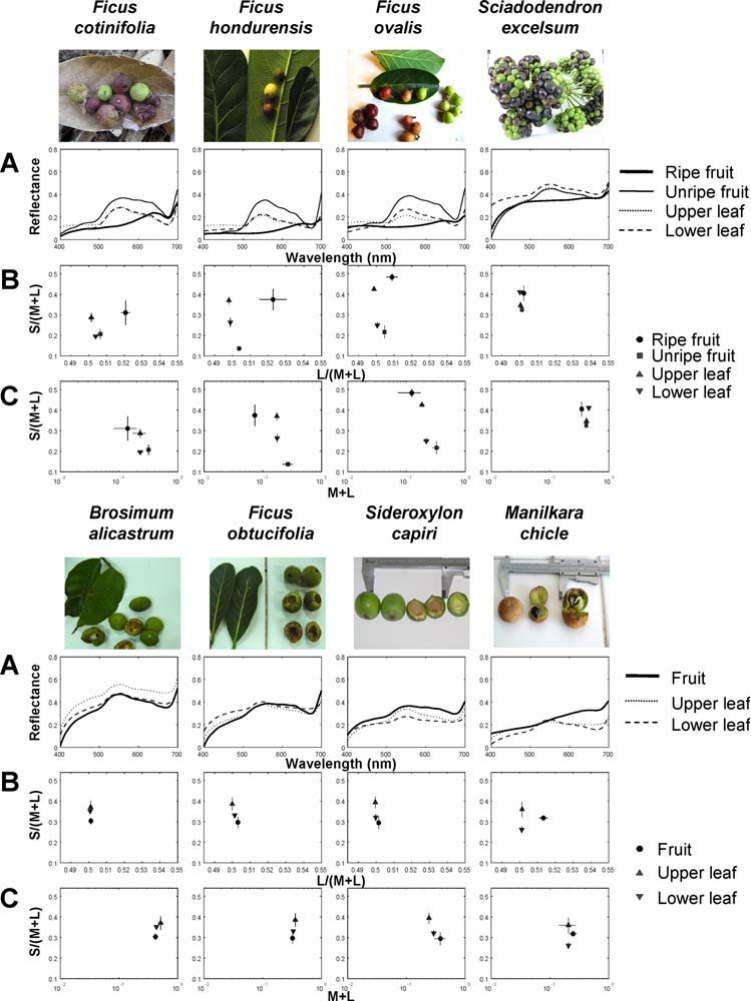
Colorimetric measurements of eight fruit species the spider monkeys frequently foraged on. (A) Reflectance spectra. (B) S/(L+M) *vs*. L/(L+M) chromaticity diagrams. (C) S/(L+M) chromaticity *vs*. L+M luminance diagrams. For fruit species which change color as they mature from greenish to reddish (*Ficus cotinifolia*, *F*. *hondurensis* and *F*. *ovalis*) or to purple (*Sciadodendron excelsum*), both ripe and unripe samples are shown in the diagrams. For fruit species remaining green (*Brosimum alicasutrum*, *F*. *obtucifolia* and *Sideroxyron capiri*) and those remaining brown (*Manilkara chicle*) throughout ripening, distinction of ripeness is not given. Data of both upper and lower sides of leaves are plotted for all species. Mean±SD for five samples are given to each data point.

Among 33 fruit species we observed spider monkeys to consume during the observation period (Table S1), 29 species were subjected to colorimetric measurement for reflectance. The reflectance spectra of eight representative species that were consumed most often and for which over 250 incidences of foraging attempts were recorded are shown in Figure 2A. Three species (*Ficus cotinifolia*, *F*. *hondurensis* and *F*. *ovalis*) of the eight have a color change from green to reddish (red, orange and yellow) during ripening, and one (*Sciadodendron excelsum*) turns from green to dark purple as it matures. Other three species (*Brosimum alicastrum*, *F*. *obtucifolia* and *Sideroxyron capiri*) stay green and the last one (*Manilkara chicle*) stays brown during maturation. *Sciadodendron excelsum* is unique among the eight species in that fruits are bunched and that ripe purple fruits are intermingled with unripe greenish fruits in the same bunch (Figure 2). The species is also unique in the sense that purple (ripe) fruits are more lustrous than green (unripe) fruits.

**Figure 3:**
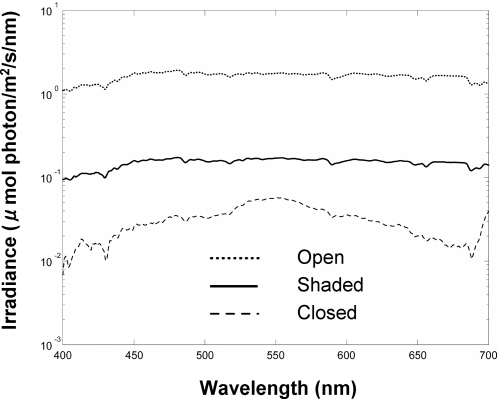
Typical irradiance spectra of illumination light in the study site forest. Open: taken under an open canopy in an overcast day. Shaded: taken under a forest shade in an overcast day. Closed: taken under dense foliage where no direct passage of sunlight reached to the ground in a lightly cloudy day.

**Figure 4:**
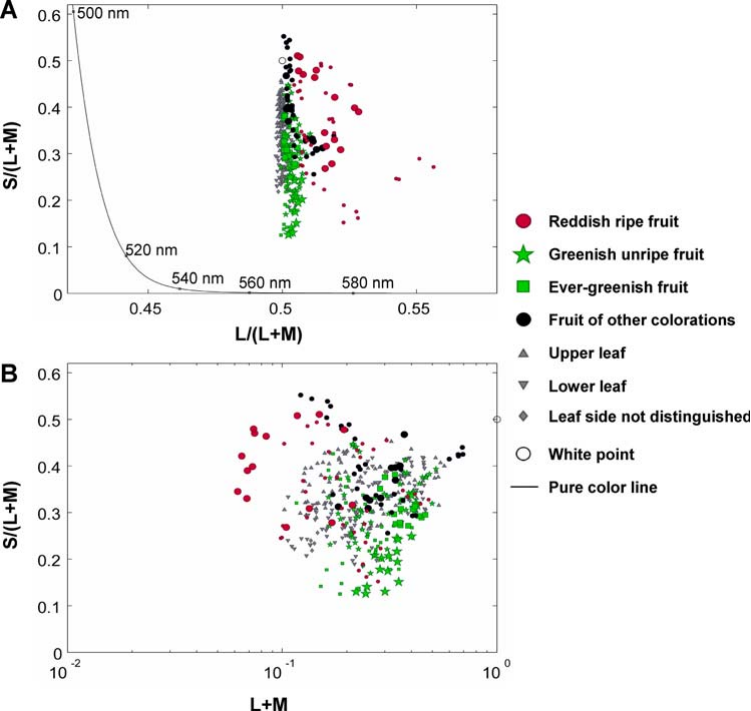
Chromaticity and luminance distribution of fruits and leaves of 29 plant species at the study site seen by trichromatic spider monkeys. (A) An L/(L+M) *vs*. S/(L+M) chromaticity diagram. The chromaticity of monochromatic light is indicated by a solid line (pure color line). (B) An L+M luminance *vs*. S/(L+M) chromaticity diagram. The luminance is shown in the logarithmic scale relative to that of a hypothetical white surface which reflects 100% of the illumination light. In both diagrams, the location of the illumination light (white point) is indicated as an open circle. The fruits which turn from greenish to reddish color (red, orange or yellow) during maturation are indicated for a reddish ripe stage (red dots) and a greenish unripe stage (green stars). For fruits which turn from green to non-reddish colors (*e.g.* purple, brown, white), unripe ones are also indicated as greenish unripe fruits (green stars). The fruits that remain green throughout ripening are indicated as ever-greenish fruits (green squares) irrespective of their ripeness. The fruits of other colorations than greenish and reddish (*e.g.* purple, brown, white) are all indicated as black dots irrespective of their ripeness. The fruit symbols for the eight species taken in Figure 2 are emphasized by their larger size. Upper and lower sides of leaves are indicated by upper-faced and lower-faced gray arrowheads, respectively. Leaf sides of *Spondias purpurea* and *Apeiba tibourbou* were not distinguished at the time of reflectance measurement and their leaves are depicted with gray diamonds. For each species and for each category of symbols, up to five data points are given corresponding to different specimens.

To assess the suitability of trichromatic vision for long-distance foraging tasks, we analyzed the chromaticity of fruits included in the spider monkey diet. We estimate the quantum catches for fruits and leaves of the 29 species, of the L (P553), M (P538), and S (P432: [8]) cone photoreceptors of the spider monkey under an illumination of forest shade (Figure 3, solid line), where the spider monkeys typically forage on fruits. Figure 4A illustrates a chromaticity diagram for these 29 species as seen by trichromatic spider monkeys, plotted in a form analogous to the MacLeod and Boynton chromaticity diagram for humans [52] consisting of L/(L+M) and S/(L+M) chromaticity axes. Figure 4B illustrates a relative luminance (represented by L+M) *versus* S/(L+M) chromaticity plot, where the luminance (sum of the quantum catch of L and M cones) is given as a relative value to the luminance of a hypothetical white surface which reflects 100% of illumination light. These diagrams depicting all 29 species together represent the case where a variety of visual objects are simultaneously viewed from a distance. It should be noted that Figure 4A is not applicable to dichromatic monkeys and that L *vs*. S/L or M *vs*. S/M chromaticity plot, instead of L+M *vs*. S/(L+M) as in Figure 4B, would represent the exact nature of chromaticity and luminance for dichromatic monkeys having only L or M opsin allele, respectively. However, the distribution of the chromaticity and luminance in the L *vs*. S/L or M *vs*. S/M plot is virtually identical to that in L+M *vs*. S/(L+M) plot, and Figure 4B can also be used for both dichromats and trichromats as has been shown in previous studies [19], [20].

It is apparent from Figure 4A that the chromaticity of leaves (both sides) is aligned vertically, occupying a narrow range of L/(L+M) values and taking a broad range of S/(L+M) values and, from Figure 4B, that the luminance of leaves ranges broadly, as a typical pattern of chromaticity and luminance of foliage [19]. The luminance distribution of leaves could be even broader, ranging over 3 log units, if measured *in situ* because of irregular patterns of local shadowing occurring continuously on the leaves [19].

The L/(L+M) values of most reddish fruit were greater than those of the leaves (Figure 4A). In contrast, the S/(L+M) chromaticity and luminance values of the reddish fruits largely overlapped with those of leaves (Figure 4A, B). On the other hand, greenish fruits largely overlapped with leaves not only in S/(L+M) and luminance but also in L/(L+M) values (Figure 4A, B). These suggest a rough consistency of perception between human and spider monkey trichromats. Although some fruits were higher or lower in S/(L+M) chromaticity than leaves, the patterns we present are largely consistent with the idea that trichromats would have an advantage for detecting reddish ripe fruits in a forest from a distance [19], [20].

When considering the situation where monkeys are close to fruits, fruits would be compared with a relatively small number of background leaves. To represent the case of close viewing, the chromaticity and luminance charts were made separately for the eight fruit species that monkeys most frequently ate (Figure 2B, C). They show that the reddish ripe fruits (*F*. *cotinifolia*, *F*. *hondurensis* and *F*. *ovalis*) can be distinguished from leaves not only by their L/(M+L) chromaticity but also by their relatively dark luminance as seen in other *Ficus* species [49]. Such local difference in luminance and also in S/(L+M) values between fruits and leaves have already been pointed out in previous studies [19], [20], but its relevance to foraging efficiency of monkeys has not been examined.

### Effect of color-vision type on foraging efficiency

**Figure 5:**
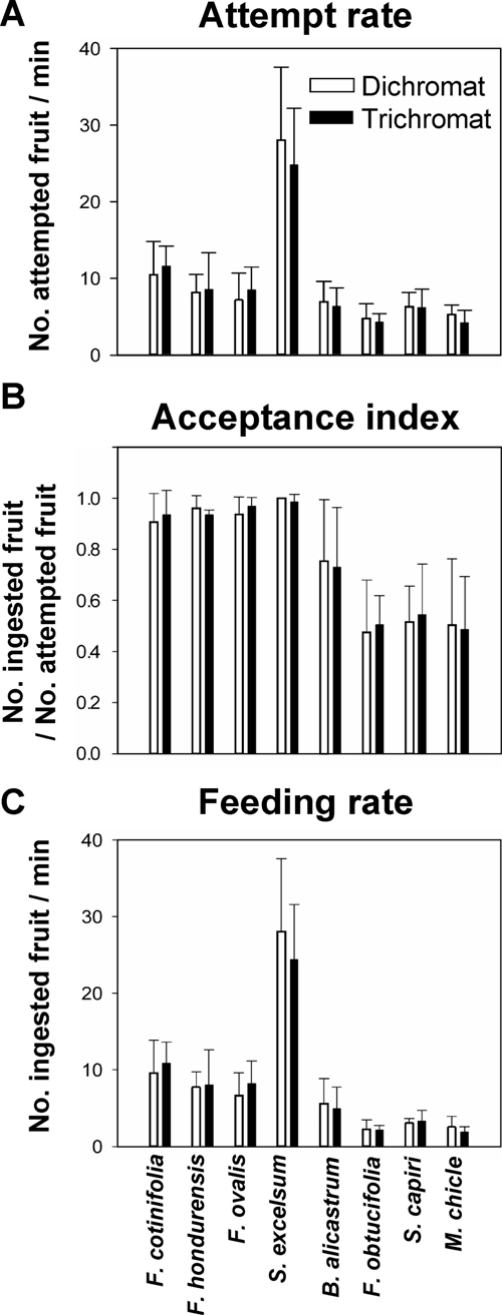
Means and SD of the three measures of foraging efficiency, attempt rate (A), acceptance index (B) and feeding rate (C) for dichromatic (open bar) and trichromatic (filled bar) monkeys for each of the eight fruit species shown in Figure 2. The number of monkeys analyzed and *p*-values in the Mann-Whitney U test for each fruit species are shown in Table 2.

We analyzed a total of 5,517 incidences of fruit selection attempts by monkeys during their visual scanning behavior (see Material and Methods for its definition) which occurred within grasping distance of the fruits. Linear mixed models (LMMs) with a covariance pattern were used to examine the effects of color-vision type on the variation of foraging efficiency while controlling for the preference of individual monkeys. No significant effects of color-vision type were found (attempt rate: *F*_1,17.5_ = 0.294, *p* = 0.595; acceptance index: *F*_1,21.3_ = 0.0001, *p* = 0.992; feeding rate: *F*_1,19.7_ = 0.177, *p* = 0.679; see Materials and Methods for definitions). We confirmed the absence of significant differences between dichromatic and trichromatic monkeys for any of the eight fruit species in any of the three measures of foraging efficiency (Figure 5; Table 2). When juveniles and/or males were excluded from the analysis, to control for age and sex effects, the results for only (sub) adult females were essentially the same.

**Table 2:** Mann-Whitney U test for comparison of foraging efficiency between dichromats and trichromats.

Species	No. of dichromat	No. of trichromat		Foraging measure		
				Attempt rate	Acceptance index	Feeding rate
*F cotinifolia*	8	8	U	27	30	26
			*p*	0.65	0.88	0.57
*F hondurensis*	4	4	U	7	5	8
			*p*	0.89	0.49	1
*F ovalis*	10	8	U	29	31	26
			*p*	0.36	0.46	0.24
*S excelsum*	5	7	U	14	12.5	14
			*p*	0.64	0.43	0.64
*B alicastrum*	6	7	U	16	11	15
			*p*	0.53	0.18	0.45
*F obtucifolia*	6	9	U	25	23	24
			*p*	0.86	0.69	0.78
*S capiri*	4	7	U	13	14	10
			*p*	0.93	1	0.53
*M chicle*	8	6	U	15	23	17
			*p*	0.28	0.95	0.41

### Determinants of variation of foraging efficiency

If visual cues are important in close range foraging, we expect that visual contrast between fruits and leaves would contribute to the monkeys' foraging efficiency. To evaluate the relative importance of red-green, blue-yellow and luminance contrasts for the three measures of the foraging efficiency, we selected the best models which consist of meaningful explanatory variables based on LMMs with a random coefficient. The luminance contrast was found to have significant positive contribution for the variations of all foraging measures for both dichromats and trichromats. Red-green and blue-yellow contrasts against the upper side of the leaf had significant negative effects for attempt rate of trichromats. As a whole, however, only the luminance contrast had consistent positive effects regardless of which leaf side (upper or lower) was included in the models (Table 3).

**Table 3:** Variables in the best LMMs explaining each foraging measure under the shaded condition.

Leaf side	Color-vision type	Foraging measure	Fixed variable	β	SE	95% C.I. of β	df	*t*	*p*	Random variable	Variance	SE
Upper	Dichromat	Attempt rate	Intercept	5.557	0.597	4.35, 6.75	45.742	9.300	<0.001	Animal ID	0.410	<0.001
			Luminace	3.566	1.374	0.78, 6.35	36.421	2.596	0.014	Error	4.426	1.057
		Acceptance index	Intercept	0.294	0.154	−0.03, 0.62	18.159	1.906	0.073	Animal ID	0.001	0.005
			Luminace	0.792	0.188	0.38, 1.20	12.522	4.216	0.001	Fruit species	0.005	0.006
			Blue-yellow	1.382	0.829	−0.35, 3.12	19.229	1.667	0.112	Error	0.033	0.010
		Feeding rate	Intercept	3.043	0.790	1.24, 4.84	8.610	3.853	0.004	Fruit species	0.003	0.005
			Luminace	6.592	1.716	2.63, 10.56	7.902	3.842	0.005	Error	0.040	0.010
	Trichromat	Attempt rate	Intercept	8.716	1.644	5.40, 12.03	43.119	5.301	<0.001	Animal ID	1.578	1.127
			Red-green	−61.885	24.750	−112.06, −11.70	36.395	−2.500	0.017	Error	3.319	0.790
			Luminace	8.723	2.299	4.06, 13.38	36.276	3.794	0.001			
			Blue-yellow	−18.672	8.489	−35.87, −1.47	36.871	−2.200	0.034			
		Acceptance index	Intercept	0.244	0.163	−0.14, 0.63	7.099	1.492	0.179	Fruit species	0.007	0.005
			Luminace	0.854	0.174	0.45, 1.25	8.146	4.901	0.001	Error	0.020	0.005
			Blue-yellow	1.555	0.916	−0.64, 3.75	6.554	1.697	0.136			
		Feeding rate	Intercept	2.656	0.817	0.75, 4.56	7.564	3.250	0.013	Animal ID	0.998	0.951
			Luminace	8.144	1.817	3.96, 12.33	8.086	4.483	0.002	Fruit species	0.858	0.881
										Error	3.726	1.015
Lower	Dichromat	Attempt rate	Intercept	5.589	0.607	4.35, 6.83	30.964	9.206	<0.001	Animal ID	0.409	0.719
			Luminace	3.092	1.251	0.55, 5.64	32.817	2.472	0.019	Error	4.576	1.181
		Acceptance index	Intercept	0.557	0.089	0.35, 0.77	6.692	6.294	<0.001	Fruit species	0.015	0.012
			Luminace	0.465	0.189	0.02, 0.91	7.077	2.463	0.043	Error	0.035	0.009
		Feeding rate	Intercept	3.385	0.948	1.18, 5.59	7.689	3.572	0.008	Animal ID	1.004	0.851
			Luminace	5.116	1.899	0.60, 9.63	6.834	2.694	0.032	Fruit species	1.554	1.248
										Error	3.083	0.921
	Trichromat	Attempt rate	Intercept	5.567	0.670	4.07, 7.07	9.768	8.305	<0.001	Animal ID	1.632	1.179
			Luminace	6.354	1.635	2.49, 10.21	7.054	3.887	0.006	fruit	0.089	0.336
			Red-green	−28.044	19.043	−72.03, 15.94	7.926	−1.473	0.179	Error	3.384	0.888
		Acceptance index	Intercept	0.522	0.083	0.32, 0.72	6.986	6.267	<0.001	Fruit species	0.016	0.010
			Luminace	0.555	0.178	0.14, 0.97	7.673	3.120	0.015	Error	0.020	0.005
		Feeding rate	Intercept	2.820	0.887	0.81, 4.83	8.812	3.180	0.011	Animal ID	1.186	1.007
			Luminace	6.958	1.773	2.87, 11.04	8.046	3.925	0.004	Fruit species	1.185	0.981
										Error	3.599	0.940

## Discussion

We determined the variation of absorption spectra of the L-M opsin alleles of a group of free-ranging spider monkeys, and measured the chromaticity and luminance of their fruit diets and background leaves as well as their close-range fruit foraging behaviors to achieve a novel, integrative study examining the utility of color vision to a natural population of New World monkeys. We found that the luminance contrast is the variable with the most explanatory power of the variation in foraging efficiency among fruit species. Consistently, there was no significant difference in foraging efficiency between dichromats and trichromats.

A classical hypothesis on color vision evolution predicts that the red-green contrast is important for primates to detect objects from a distance against a background of foliage because foliages show a narrow range of L/(L+M) chromaticity while showing a broad range of S/(L+M) and luminance values which are likely to be distractive to the task of detecting objects [19], [20], [28]. The distribution of chromaticity and luminance of fruits and leaves at our study site was largely consistent with this prediction (Figure 4). It also predicts that visual cues would overall be less important in close-range foraging attempts than in detecting resources from a distance because other sensory cues, such as scent, would be available in short-range foraging. Contrary to the prediction, our observation of foraging behaviors suggest that visual contrasts, especially the luminance contrast, can serve as an important cue in foraging attempts at close range, where fruits would be compared with relatively small number of background leaves and local difference in luminance and blue-yellow values could be used as effective cues. Indeed, we frequently observed male monkeys (*i.e.* dichromats) picking reddish fruits among greenish ones seemingly with no difficulty. This is not to undermine the importance of olfaction in food selection, which we did not measure in this study, but rather to emphasize the sustained importance of visual contrasts, even at short distances.

Our finding of the relatively higher importance of luminance contrast than red-green contrast during close-range foraging may also seem incongruent with the prediction from a study of chromatic and luminance sensitivity of humans to different spatial frequency modulations of natural images [24]. This study predicted that the primate color vision would be efficient at encoding images of reddish or yellowish fruit against a leafy background at viewing distances commensurate with a typical grasping distance of about 40 cm (*i.e.* at a low spatial frequency). The human visual system shows a greater contrast sensitivity to chromatic gratings than to luminance gratings at low spatial frequencies (below 0.5 cycles/deg) but shows a greater contrast sensitivity to luminance than to chromatic gratings at high spatial frequencies (above 0.5 cycles/deg) [23]. The viewing distance at the foraging attempts observed in this study was about 30 to 40 cm (approximately an arm length of spider monkeys), and diameters of frequently consumed fruits ranged from 1 to 3 cm (Table S1). This corresponds to 0.18 to 0.7 fruits/deg (86 to 343 min of arc/fruit) for which both chromatic and luminance contrasts would work well [23]. The luminance contrast is known to play a predominant role in detecting contour and measuring depth [53], [54] and, whenever available, may be exploited as an essential component of visual information in the detection of fruits.

Fruit foraging behaviors can be conceptually divided into three stages: stage 1 refers to detection, stage 2 to inspection, and stage 3 to ingestion. It should be noted that all of our three measures of foraging efficiency concern stage 1. The attempt rate indirectly evaluates how frequently fruits were detected, through our observation of monkeys' inspection behaviors at stage 2 (*i.e.* visual inspection, smelling, touching and/or biting). The acceptance index evaluates how accurately a monkey selected an edible fruit in the detection at stage 1 prior to the inspection behaviors at stage 2. If a fruit was rejected after inspection, we interpret that the initial selection for edible fruit at stage 1 was inaccurate. The feeding rate evaluates how efficiently the initial detection at stage 1 resulted in unit-time food intake. Therefore, our correlation analyses evaluate, through the three indices, how important the visual cues are during the stage 1 of foraging.

The high acceptance index for the reddish fruits (*F*. *cotinifolia*, *F*. *hondurensis* and *F*. *ovalis*) (Figure 5B) may exhibit a ceiling effect so that exact difference of the acceptance index between dichromats and trichromats may have remained concealed. However, this also implies that the luminance contrast of these fruits was high enough to override the possible advantage of trichromats benefited from the red-green contrast. It is important to point out that these fruits comprised a predominant component (54% of total fruit attempts) of the diet of spider monkeys at the study site.

Various sensory modalities can be involved in the stages 1 and 2 and the specific senses most important in the two stages can be different. Our finding of positive contributions of the luminance contrast to three measures of foraging efficiency supports the importance of vision during stage 1, although contribution of other sensory cues (such as odors) remain to be examined, which will be a difficult undertaking. This study did not evaluate the contribution of vision in stage 2, which could be studied by examining proportion of the vision-dependent rejection events against total rejection events, in which monkeys closely looked at a fruit then left it. This is one of our future research foci.

A recent field study of fruit foraging behavior of capuchin monkeys showed no statistical difference between trichromats and dichromats in feeding and energy intake rates [36], which is consistent with our present observation for spider monkeys. The capuchin study, however, did not include investigation of spectral properties of the fruit targets. Our findings regarding spider monkeys increase our understanding a step further by providing a logical basis for the absence of differences between trichromats and dichromats: because luminance cues in dichromatic and trichromatic phenotypes are similar, trichromatic and dichromatic monkeys are expected to be similar in their ability to detect fruits.

Field observations of foraging behaviors of New World monkeys have thus far either demonstrated dichromat advantage for insect foraging [43] or failed to detect advantage of trichromats in fruit foraging ([36]; present study). This leaves a fundamental question unanswered regarding what maintains trichromatic vision in primate populations, because trichromacy (*i.e.* heterozygosity on the L-M opsin alleles) would have disappeared without a selective force acting to maintain allelic variations of the L-M opsin. The trichromat advantage could indeed be present in fruit foraging but we have not yet been able to identify it. One such possibility is the importance of trichromacy for long-distance resource detection as is predicted from our colorimetric measurement of fruits and leaves at the study site (Figure 4).

Future research endeavors should focus on long-distance foraging success by, for example, noting the first individual to arrive at the fruiting tree or fruiting bough, although the predicted advantage of trichromats in detecting reddish fruits from a distance is not necessarily apparent because monkeys may have a mental “map” of the locations of fruiting trees in their foraging area [55], [56]. In fact, studies of wild tamarins have detected no clear effect of color-vision type (dichromatic or trichromatic) or sex on the individuals leading the group to feeding trees [34], [35]. It is also important to collect behavioral data under relatively dim conditions, such as under the most dense (‘closed’) canopy or at dawn and dusk because modeling and psychophysical studies have predicted an advantage on trichromats in dim light conditions for chromatic discrimination [48], [57]. However, this will be difficult given the poor visibility of monkeys to observers. Our data were taken mostly under shaded canopy conditions, in which the foliage was not sufficiently dense to completely block the passage of direct sunlight. More data are also desired from all varieties of foliage density, height in canopy, weather, time of a day, and season. In particular, more data need to be collected in the dry season when fruits run short and competition for them may increase [28], [58]. For the fruit species that remain green throughout ripening, reflectance data of ripe and unripe fruit samples need to be collected, which would enable us to examine correlation of foraging efficiency with visual contrast between ripe and unripe fruits of various plant species. While recording foraging behaviors, more detailed information on fruit phenology (*e.g.* ripe/unripe ratio, size and density in a tree) and background substrates (*e.g.* sides of leaves) would also be helpful. Extensions of the field study to other groups and species is also desirable given the spectral characterization of likely different repertoires of fruit diets as shown in other study sites of spider monkeys in Costa Rica [38]. Observation could also be directed to other food items including leaves and flowers, and to predator detection and receiving social signals [22], [59], [60]. Finally, with a larger data set, degree of differences in behavioral performances between phenotypes could be more confidently quantified in every environmental condition, which will allow us to find not only trichromatic but also dichromatic advantages in various environmental settings.

Our study presented a novel methodology and conceptual framework to evaluate the behavioral significance of color vision in primates and shed new light on the importance of visual, especially luminance, contrast during short-range foraging on fruits. This also demonstrated an effective interdisciplinary approach to open the door for exciting prospects of future research into the evolutionary significance of primate color vision.

## Materials and Methods

### Study site and animals

The field study was carried out in Santa Rosa National Park of the Area de Conservación Guanacaste (ACG), in north-western Costa Rica (10°45′–11°00′N, 85°30′–85°45′W). The dominant habitat in Santa Rosa is tropical dry forest in which the majority of the understory plants and nonriparian trees lose their leaves in the dry season (January to mid May). The canopy height of the forest rarely exceeds 30 m [44], [61].

We studied a group of black-handed spider monkeys (*Ateles geoffroyi frontatus*) consisting of approximately 20 individuals, with 1 adult and 2–3 sub-adult males, ∼5 adult and ∼5 sub-adult females and several juveniles and infants at any given time. Behavioral observations were carried out in separate periods during June 2004–September 2005 (five months in the wet and three months in the dry season). Variation in group size is a result of immigration and emigration of group members during the study period. Table 1 shows the total number of the monkeys for which behavioral data were collected. All monkeys were individually identified based on a combination of their age, sex, body size, facial markings and pelage patterns.

### Reconstitution of visual pigments

Fecal samples were collected from individually identified monkeys in the study site. DNA was isolated from the fecal samples, and the L-M opsin gene regions were PCR-amplified and sequenced as previously described [13]. Two alleles of the L-M opsin gene were previously identified from the study animals, one (P560) as having Ser, Tyr and Thr at the amino acid sites 180, 277 and 285, respectively, with an expected λ_max_ value at 560 nm, and the other (P552) as having Ser, Phe and Thr with an expected λ_max_ value at 552 nm, on the basis of the three-site rule of the primate L-M opsin genes [62], [63]. The opsin cDNAs with deduced amino acid sequences identical to those of the two alleles were created by the site-directed mutagenesis as in Hiramatsu et al. (2005) [13] using the squirrel monkey P560 opsin cDNAs as the template which was previously synthesized [62]. Photopigments were reconstituted *in vitro* by the transient expression system with cultured COS 1 cells using these cDNAs and 11-*cis* retinal as previously described [13]. The previously named P560 and P552 of spider monkeys [13] correspond to the P553 and P538 of this study, respectively.

### Calculation of quantum catch

The quantum catches of L, M and S cones, containing the visual pigment P553, P538 and P432, respectively, were calculated from the following formula [19], [48],
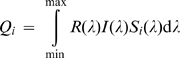
where *Q_i_* is the quantum catch of a photoreceptor *i* (*i* = L, M, S), λ is the wavelength, and ‘min’ and ‘max’ are the lower and upper limits of the visible spectrum respectively. Here, we assumed ‘min’ = 400 nm and ‘max’ = 700 nm. *R*(λ) is the reflectance spectrum of a stimulus, *I*(λ) is the illumination spectrum, and *S_i_*(λ) is the spectral sensitivity function of the *i*-th photoreceptor.

The reflectance spectra *R*(λ)of fruits and their background leaves were measured with five or more of representative samples respectively by using the USB2000 miniature fiber optic spectrometer (Ocean Optics, Dunedin, FL). For the measurement, a 3100 K tungsten halogen lamp (LS-1, Ocean Optics) was used as a light source with a reflection probe (R400-7-UV/VIS, Ocean Optics) and a white reflectance standard (WS-1, Ocean Optics).

The illumination spectra *I*(λ) in the forest were measured as the absolute irradiance spectra using the USB2000 spectrometer calibrated with a 3100 K tungsten halogen calibration light source (LS-1-CAL, Ocean Optics). The spectra were taken through a cosine corrector (CC-3, Ocean Optics) attached to an optical fiber (QP200-2-UV/VIS, Ocean Optics).

The spectral sensitivity function *S_i_*(λ) of each of the three cone photoreceptors L, M and S was estimated by considering the filtering effects of the lens and macular pigment [19]. We first estimated the absorbance spectra of each of the three visual pigments (*S_pigment_*) using the modified version of Lambs' equation [64] by giving the λ_max_ value of each visual pigment to the equation. Next, we estimated the absorption rate spectra of each pigment at retina (*S_retina_*) by the formula, *S_retina_*(λ) = 1−10^−*aSpigment*(λ)^, where *a* is the longitudinal optical density of cones to axial illumination at λ_max_ and is assumed to be 0.3 by following a previous study [19]. We then estimated the absorption rate spectra of each pigment at the cornea (*S_cornea_*) by the formula, *S_conea_*(λ) = 10^−[*Lens*(λ)+*Macular*(λ)]^×*S_retina_*(λ), where *Lens*(λ) and *Macular*(λ) represent the optical density spectra of lens and macular pigment, respectively, measured in humans [65]. Finally, the spectral sensitivity functions *S_i_*(λ) of the three cones were obtained by normalizing the *S_cornea_* functions so that the quantum catch of the three receptors under a given illumination were all set to be 1 for the light emitted from a hypothetical white surface which reflects 100% of the illumination light. This normalization was carried out based on the assumption of color constancy under which we assume that the monkeys' eyes would adapt to the varying spectral compositions of illumination light so that the illumination light appears white (colorless) presumptively.

### Estimation of chromaticity and luminance contrasts

For trichromatic primates, perceived color, in terms of chromaticity, can be described as a ratio of the quantum catch among their L, M and S cones and expressed as a point in a diagram analogous to the MacLeod-Boynton diagram [52] consisting of L/(L+M) and S/(L+M) axes, where the former represents a ratio of quantum catch of L cones to that of L and M cones and the latter represents that ratio for S cones to L and M cones. Thus, the chromaticity values, L/(L+M) and S/(L+M), of each object were given as *Q*_L_/(*Q*_L_+*Q*_M_) and *Q*_S_/(*Q*_L_+*Q*_M_) values, respectively. The relative luminance value, L+M, of each object was estimated by dividing its *Q*_L_+*Q*_M_ value by that of a hypothetical white surface that reflects 100% of the given illumination light.

The contrast for each cone channel was defined as Δ*f_i_* = |ln(*Q* ^f^ *_i_*)−ln(*Q* ^b^ *_i_*)|, where *Q* ^f^ *_i_* and *Q* ^b^ *_i_* denote the quantum catches of the receptor *i* (*i* = L, M, S) for fruit and background, respectively [66], [67]. The luminance contrast, Δ*L*, was assumed to be the contrast of L or M cones between fruit and background and was given as Δ*L*_d_ = Δ*f*_L_ or Δ*f*_M_ for the two dichromatic phenotypes and as Δ*L*_t_ = |ln(*Q* ^f^_M_+*Q* ^f^_L_)−ln(*Q* ^b^_M_+*Q* ^b^_L_)| for the trichromatic phenotype. The blue-yellow contrast was defined as *BY*_d_ = |Δ*f*_L_−Δ*f*_S_| or |Δ*f*_M_−Δ*f*_S_| for dichromats and as *BY*_t_ = |Δ*L*_t_−Δ*f*_S_| for trichromats. The red-green contrast, applicable only for trichromats, is defined as *RG* = |Δ*f*_L_−Δ*f*_M_|.

### Behavioral observation

Spider monkeys travel quickly, have large home ranges and are highly arboreal, spending much of their time in the highest levels of the canopy [68]–[70]. We conducted short (1–5 min) continuous focal animal samples while the monkeys were feeding in fruit trees [71]. Sample durations were kept short in order to observe as many group members as possible in the same tree before the group moved on and because poor visibility conditions precluded lengthy samples. Spider monkeys live in communities that are characterized by a high degree of fission-fusion dynamics, in which members frequently fission and fuse into subgroups consisting of variable size and composition repeatedly during the day [72]. We followed the first subgroup encountered in a day and chose focal animals randomly from the subgroup members. Focal samples were collected under a variety of environmental conditions distinguished by foliage density, height in canopy, weather, time of a day, and season.

By extension of Smith et al. (2003) [31], we conceptually divided foraging behaviors on fruit into three stages: (1) detection of fruits, (2) inspection of fruits by close visual scrutiny or by using other sensory modalities (*i.e.* smell, touch and taste), and (3) ingestion of fruits. An observer cannot definitively know when a monkey has detected a fruit (stage 1). Therefore, by the actions monkeys made in stage 2, we indirectly knew that the stage 1 has occurred.

We calculated the ‘attempt rate’ as the total number of fruits investigated per total duration of scanning behavior (min) in a fruiting tree for every monkey-fruit species combination. We define scanning as a foraging behavior in which the monkey's eyes are directed towards and scan over substrates in the nearby foliage while either locomoting or stationary in a tree and is distinguished from general vigilance, which we recorded if the monkey scans over a wider area of the environment with increased range of head movement. Next, we considered the total number of ingested fruits (at stage 3) out of the total number of investigated fruits (at stage 2) for every monkey-fruit species combination, as the ‘acceptance index’. Finally, we calculated the ‘feeding rate’ as the total number of ingested fruit divided by the total duration (min) of scanning behavior for every monkey-fruit species combination. We regarded the three indices as complementary measures of foraging efficiency.

For the fruit species for which human observers can discern ripe and unripe states by their change of color from greenish to reddish or purple, we included only foraging attempts in those trees where both ripe and unripe fruits were present, although exact proportions of ripe fruits were not examined. We excluded monkeys from our analyses for which we did not have at least 10 fruit investigations recorded for the species in a given condition.

Linear mixed models (LMMs) with covariance pattern with animal ID as a random factor were used to examine the effects of color-vision type on the variation of foraging efficiency. LMMs allow both fixed and random variables to be fitted to a model. The inclusion of random variables allowed us to model residual correlations due to the repeated observations of the same individual [73]. Color-vision type (dichotomous: dichromat = 0, trichromat = 1) and fruit species (nominal: *B. alicastrum*, *F. cotinifolia*, *F. hondurensis*, *F. obtucifolia*, *F. ovalis*, *M. chicle S. capiri* and *S. excelsum*) were included in models as fixed factors and each foraging measure was selected as a continuous dependent variable. Maximum-likelihood methods were used for model estimation.

To compare the measures of foraging efficiency between trichromats and dichromats for each fruit species, we used the Mann-Whitney U test. P538 dichromats and P553 dichromats were not distinguished in this study because of the small sample size of P538 dichromats (3 individuals; see Table 1).

LMMs with random coefficients were used to estimate the contribution of each visual contrast to the foraging efficiency of dichromats and trichromats respectively. We considered the 3,117 incidences of fruit feeding attempts that occurred under the forest shade condition and that were directed towards seven of the eight most-eaten species of fruit. We excluded the incidences at the open canopy condition because fruits were often devoid of leafy background in this condition. We excluded *S. excelsum* incidences because the backgrounds for these fruits were usually unripe fruits and the visual contrast was between ripe and unripe fruits intermingled in the same bunch (Figure 2). Therefore, this species was not appropriate for this analysis as our priority is the evaluation of ripe fruits viewed against a leafy background. Each foraging measure (attempt rate, acceptance index and feeding rate) was entered as a continuous dependent variable, and animal ID and fruit species were entered as nominal random factors in each initial model. For each foraging measure, two analyses were conducted including red-green, blue-yellow and luminance contrasts of fruits against either the upper or the lower side of the leaf as continuous fixed factors. The best model was selected based on Akaike's information criterion (AIC), which compares the adequacy of several models and identifies the model that best explains the variance of the dependent variable as that with the lowest AIC value [74], [75]. Random variables were excluded from the best model when the variance component was estimated to be zero. Maximum-likelihood methods were used for model estimation. All analyses were carried out using SPSS (version 15.0, SPSS Inc).

## Supporting Information

Table S1 The 33 fruit species consumed by spider monkeys at the study site during the observation period.
(0.11 MB PDF)

## References

[1] Jacobs GH (1996). Primate photopigments and primate color vision.. Proc Natl Acad Sci U S A.

[2] Mollon JD, Bowmaker JK, Jacobs GH (1984). Variations of colour vision in a New World primate can be explained by polymorphism of retinal photopigments.. Proc R Soc Lond B.

[3] Kawamura S, Hirai M, Takenaka O, Radlwimmer FB, Yokoyama S (2001). Genomic and spectral analyses of long to middle wavelength-sensitive visual pigments of common marmoset (*Callithrix jacchus*).. Gene.

[4] Jacobs GH (1998). A perspective on color vision in platyrrhine monkeys.. Vision Res.

[5] Jacobs GH, Deegan JF, Neitz J, Crognale MA, Neitz M (1993). Photopigments and color vision in the nocturnal monkey, *Aotus*.. Vision Res.

[6] Levenson DH, Fernandez-Duque E, Evans S, Jacobs GH (2007). Mutational changes in S-cone opsin genes common to both nocturnal and cathemeral *Aotus* monkeys.. Am J Primatol.

[7] Jacobs GH, Neitz M, Deegan JF, Neitz J (1996). Trichromatic colour vision in New World monkeys.. Nature.

[8] Jacobs GH, Deegan JF (2001). Photopigments and colour vision in New World monkeys from the family Atelidae.. Proc R Soc Lond B.

[9] Jacobs GH, Deegan JF (2005). Polymorphic New World monkeys with more than three M/L cone types.. J Opt Soc Am A.

[10] Talebi MG, Pope TR, Vogel ER, Neitz M, Dominy NJ (2006). Polymorphism of visual pigment genes in the muriqui (Primates, Atelidae).. Mol Ecol.

[11] Jacobs GH (2007). New World monkeys and color.. Int J Primatol.

[12] Surridge AK, Smith AC, Buchanan-Smith HM, Mundy NI (2002). Single-copy nuclear DNA sequences obtained from noninvasively collected primate feces.. Am J Primatol.

[13] Hiramatsu C, Tsutsui T, Matsumoto Y, Aureli F, Fedigan LM (2005). Color-vision polymorphism in wild capuchins (*Cebus capucinus*) and spider monkeys (*Ateles geoffroyi*) in Costa Rica.. Am J Primatol.

[14] Kolb H, Dekorver L (1991). Midget ganglion cells of the parafovea of the human retina: a study by electron microscopy and serial section reconstructions.. J Comp Neurol.

[15] Yamada ES, Silveira LC, Perry VH (1996). Morphology, dendritic field size, somal size, density, and coverage of M and P retinal ganglion cells of dichromatic Cebus monkeys.. Vis Neurosci.

[16] Dacey DM, Lee BB (1994). The ‘blue-on’ opponent pathway in primate retina originates from a distinct bistratified ganglion cell type.. Nature.

[17] Silveira LC, Lee BB, Yamada ES, Kremers J, Hunt DM (1999). Ganglion cells of a short-wavelength-sensitive cone pathway in New World monkeys: morphology and physiology.. Vis Neurosci.

[18] Regan BC, Julliot C, Simmen B, Vienot F, Charles-Dominique P (1998). Frugivory and colour vision in *Alouatta seniculus*, a trichromatic platyrrhine monkey.. Vision Res.

[19] Sumner P, Mollon JD (2000). Catarrhine photopigments are optimized for detecting targets against a foliage background.. J Exp Biol.

[20] Regan BC, Julliot C, Simmen B, Vienot F, Charles-Dominique P (2001). Fruits, foliage and the evolution of primate colour vision.. Phil Trans R Soc Lond B.

[21] Sumner P, Mollon JD (2003). Colors of primate pelage and skin: objective assessment of conspicuousness.. Am J Primatol.

[22] Fernandez AA, Morris MR (2007). Sexual selection and trichromatic color vision in primates: statistical support for the preexisting-bias hypothesis.. Am Nat.

[23] Mullen KT (1985). The contrast sensitivity of human colour vision to red-green and blue-yellow chromatic gratings.. J Physiol.

[24] Parraga CA, Troscianko T, Tolhurst DJ (2002). Spatiochromatic properties of natural images and human vision.. Curr Biol.

[25] Lucas PW, Darvell BW, Lee PKD, Yuen TDB, Choong MF (1998). Colour cues for leaf food selection by long-tailed macaques (*Macaca fascicularis*) with a new suggestion for the evolution of trichromatic colour vision.. Folia Primatol.

[26] Lucas PW, Dominy NJ, Riba-Hernandez P, Stoner KE, Yamashita N (2003). Evolution and function of routine trichromatic vision in primates.. Evolution.

[27] Dominy NJ, Lucas PW (2004). Significance of color, calories, and climate to the visual ecology of catarrhines.. Am J Primatol.

[28] Dominy NJ, Lucas PW (2001). Ecological importance of trichromatic vision to primates.. Nature.

[29] Riba-Hernandez P, Stoner KE, Lucas PW (2005). Sugar concentration of fruits and their detection via color in the Central American spider monkey (*Ateles geoffroyi*).. Am J Primatol.

[30] Caine NG, Mundy NI (2000). Demonstration of a foraging advantage for trichromatic marmosets (*Callithrix geoffroyi*) dependent on food colour.. Proc R Soc Lond B.

[31] Smith AC, Buchanan-Smith HM, Surridge AK, Osorio D, Mundy NI (2003). The effect of colour vision status on the detection and selection of fruits by tamarins (*Saguinus* spp.).. J Exp Biol.

[32] Caine NG, Miller LE (2002). Seeing red: consequences of individual differences in color vision in callitrichid primates.. Eat or be eaten.

[33] Smith AC, Buchanan-Smith HM, Surridge AK, Mundy NI (2005). Factors affecting group spread within wild mixed-species troops of saddleback and mustached tamarins.. Int J Primatol.

[34] Smith AC, Buchanan-Smith HM, Surridge AK, Mundy NI (2003). Leaders of progressions in wild mixed-species troops of saddleback (*Saguinus fuscicollis*) and mustached tamarins (*S. mystax*), with emphasis on color vision and sex.. Am J Primatol.

[35] Dominy NJ, Garber PA, Bicca-Marques JC, Azevedo-Lopes MA (2003). Do female tamarins use visual cues to detect fruit rewards more successfully than do males?. Anim Behav.

[36] Vogel ER, Neitz M, Dominy NJ (2007). Effect of color vision phenotype on the foraging of wild white-faced capuchins, *Cebus capucinus*.. Behav Ecol.

[37] Melin AD, Fedigan LM, Hiramatsu C, Kawamura S (2008). Polymorphic color vision in white-faced capuchins (*Cebus capucinus*): Is there foraging niche divergence among phenotypes?. Behav Ecol Sociobiol.

[38] Riba-Hernandez P, Stoner KE, Osorio D (2004). Effect of polymorphic colour vision for fruit detection in the spider monkey *Ateles geoffroyi*, and its implications for the maintenance of polymorphic colour vision in platyrrhine monkeys.. J Exp Biol.

[39] Stoner KE, Riba-Hernandez P, Lucas PW (2005). Comparative use of color vision for frugivory by sympatric species of platyrrhines.. Am J Primatol.

[40] De Araujo MF, Lima EM, Pessoa VF (2006). Modeling dichromatic and trichromatic sensitivity to the color properties of fruits eaten by squirrel monkeys (*Saimiri sciureus*).. Am J Primatol.

[41] Saito A, Mikami A, Kawamura S, Ueno Y, Hiramatsu C (2005). Advantage of dichromats over trichromats in discrimination of color-camouflaged stimuli in nonhuman primates.. Am J Primatol.

[42] Caine NG, Surridge AK, Mundy NI (2003). Dichromatic and trichromatic *Callithrix geoffroyi* differ in relative foraging ability for red-green color-camouflaged and non-camouflaged food.. Int J Primatol.

[43] Melin AD, Fedigan LM, Hiramatsu C, Sendall C, Kawamura S (2007). Effects of colour vision phenotype on insect capture by a free-ranging population of white-faced capuchins (*Cebus capucinus*).. Anim Behav.

[44] Chapman CA (1988). Patterns of foraging and range use by three species of neotropical primates.. Primates.

[45] Asensio N, Korstjens AH, Schaffner CM, Aureli F (2008). Intragroup aggression, fission-fusion dynamics and feeding competition in spider monkeys.. Behaviour.

[46] Allen G (1879). The Color Sense: Its Origin and Development..

[47] Osorio D, Vorobyev M (1996). Colour vision as an adaptation to frugivory in primates.. Proc R Soc Lond B.

[48] Osorio D, Smith AC, Vorobyev M, Buchanan-Smith HM (2004). Detection of fruit and the selection of primate visual pigments for color vision.. Am Nat.

[49] Sumner P, Mollon JD (2000). Chromaticity as a signal of ripeness in fruits taken by primates.. J Exp Biol.

[50] Kinzey WG, Kinzey WG (1997). New World Primates: Ecology, Evolution, and Behavior..

[51] Nunes A (1995). Foraging and ranging patterns in white-bellied spider monkeys.. Folia Primatol.

[52] MacLeod DI, Boynton RM (1979). Chromaticity diagram showing cone excitation by stimuli of equal luminance.. J Opt Soc Am.

[53] Lu C, Fender DH (1972). The interaction of color and luminance in stereoscopic vision.. Invest Ophthalmol.

[54] Livingstone M, Hubel D (1988). Segregation of form, color, movement, and depth: anatomy, physiology, and perception.. Science.

[55] Garber PA, S B, Garber PA (2000). Evidence for the use of spatial, temporal and social information by some primate foragers.. On the move: how and why animals travel in groups.

[56] Di Fiore A, Fleischer RC (2004). Microsatellite markers for woolly monkeys (*Lagothrix lagotricha*) and their amplification in other New World primates (Primates: Platyrrhini).. Mol Ecol Notes.

[57] Rowe MP, Jacobs GH (2007). Naturalistic color discriminations in polymorphic platyrrhine monkeys: effects of stimulus luminance and duration examined with functional substitution.. Vis Neurosci.

[58] Dominy NJ, Svenning JC, Li WH (2003). Historical contingency in the evolution of primate color vision.. J Hum Evol.

[59] Vorobyev M (2004). Ecology and evolution of primate colour vision.. Clin Exp Optom.

[60] Changizi MA, Zhang Q, Shimojo S (2006). Bare skin, blood and the evolution of primate color vision.. Biol Lett.

[61] Janzen DH (1986). Guanacaste national park: tropical ecological, and cultural restoration..

[62] Hiramatsu C, Radlwimmer FB, Yokoyama S, Kawamura S (2004). Mutagenesis and reconstitution of middle-to-long-wave-sensitive visual pigments of New World monkeys for testing the tuning effect of residues at sites 229 and 233.. Vision Res.

[63] Yokoyama S, Radlwimmer FB (2001). The molecular genetics and evolution of red and green color vision in vertebrates.. Genetics.

[64] Govardovskii VI, Fyhrquist N, Reuter T, Kuzmin DG, Donner K (2000). In search of the visual pigment template.. Visual Neurosci.

[65] Wyzecki G, Stiles WS (1982). Color science: concepts and methods, quantitative data, and formulae..

[66] Vorobyev M, Osorio D, Bennett ATD, Marshall NJ, Cuthill IC (1998). Tetrachromacy, oil droplets and bird plumage colours.. J Comp Physiol A.

[67] Vorobyev M, Brandt R, Peitsch D, Laughlin SB, Menzel R (2001). Colour thresholds and receptor noise: behaviour and physiology compared.. Vision Res.

[68] Campbell CJ, Aureli F, Chapman CA, Ramos-Fernandez G, Matthews K (2005). Terrestrial behaviour of *Ateles* spp.. Int J Primatol.

[69] van Roosemalem MGM (1985). Habitat preferences, diet, feeding strategy and social organization of the black spider monkey (*Ateles paniscus paniscus* Linnaeus 1758) in Suriname.. Acta Amazon.

[70] Chapman CA, Chapman LJ, McLaughlin RL (1989). Multiple central place foraging by spider monkeys: travel consequences of using many sleeping sites.. Oecologia.

[71] Altmann J (1974). Observational study of behavior: sampling methods.. Behaviour.

[72] Aureli F, Schaffner CM, Campbell CJ (2008). Social interactions, social relationships and the social system of spider monkeys.. Spider monkeys: behavior, ecology and evolution of the genus *Ateles*.

[73] Brown H, Prescott R (1999). Applied mixed models in medicine..

[74] Pinheiro JC, Bates DM (2000). Mixed effects models in S and S-Plus..

[75] Tabachnick BG, Fidell LS (2007). Using multivariate statistics..

