# Supplementary material for: Importance of Achromatic Contrast in Short-Range Fruit Foraging of Primates
Source: PLoS One. 2008 Oct 6;3(10):e3356. doi: 10.1371/journal.pone.0003356 (PMC2559900; doi:10.1371/journal.pone.0003356)
Supplement: Table S1 — The 33 fruit species consumed by spider monkeys at the study site during the observation period. (0.11 MB PDF) [file pone.0003356.s001.pdf]

Table S1. The 33 fruit species consumed by spider monkeys at the study site during the observation period

Family	Species	Mature color	Immature color	Color normally consumed	Shape	Maximum diameter (cm)§
Anacardiaceae	*Spondias mombin* L.	yellow	green	yellow	round	2.5-3
	Spondias purpurea L.	orange/red	green	orange/red	round	1.5-2
Annonaceae	*Annona reticulata* L.	light brown	green	light brown	round	5
Apocynaceae	*Stemmadenia obovata* Schum.	green†	green	green	oval	5
Araliaceae	***Sciadodendron excelsum*** **Griseb.**	dark purple	green	dark purple	round	0.7-1
Asclepiadaceae	*Marsdenia engleriana* W. Rothe	green	green	green	oval	10-15
Bombacaceae	*Bombacopsis quinata* Dugand*	light brown	green	light brown	cylindrical	5
	Pseudobombax septinatum Dugand*	green	green	green	oval	10-15
Boraginaceae	*Cordia panamensis* Riley	white	green	white	round	0.7-1
Burseraceae	*Bursera simaruba* Sarg.	green‡	green	green	round	1
Clusiaceae	*Garcinia intermedia* Hammel*	orange	green	orange	round	1.5
Ebenaceae	*Diospyros costaricensis* Provance & A. C. Sanders	orange	green	orange	round	1.5-2
Euphorbiaceae	*Sapium glandulosum (* L) Morong	brown¶	green	green	round	1
Malpighiaceae	*Bunchosia biocellata* Schlecht.	red	green	red	round	1.5
Moraceae	***Brosimum allicastrum*** **Swartz.**	green	green	green	round	2.5-3
	Castilla elastica Cerv.	brown¶	green	brown¶	compound discoid	1
	Cecropia peltata L.	green	green	green	cylindrical	5
	Ficus cotinifolia **H.B.K.**	red	green	red	round	1
	Ficus goldmanii Standal.	green	green	green	round	1-1.5
	Ficus hondurensis **Standal.**	red	green	red	round	1
	Ficus morazaniana Burger.	green	green	green	round	1.5-2
	Ficus obtusifolia **Kunth**	green	green	green	round	2-2.5
	Ficus ovalis **Miq.**	red	green	red	round	1
	Maclura tinctoria Don	green	green	green	round	1.5-2
	Sideroxylon capiri **Pittier**	green	green	green	round	2.5-3
Polygonaceae	*Coccoloba guanacastensis* W. C. Burger*	dark purple	green	dark purple	round	1
Rhamnaceae	*Karwinskia caldronii* Standl.	dark purple	green	dark purple	round	1
Rubiaceae	*Genipa americana* L.	light brown	green¶	light brown	round	4-5
	Guettarda macrosperma Donn. Sm.	red	green	red	round	1.5-2
Sapindaceae	*Allophylus occidentalis* Radlk.	red/yellow	green¶	red/yellow	round	0.5-1
Sapotaceae	***Manilkara chicle*** **Gilly**	light brown	light brown	light brown	round	2.5-3
Simaroubaceae	*Simarouba glauca* DC.	purple	green	purple	round	1-1.5
Tiliaceae	*Apeiba tibourbou* Aubl.	dark brown	green	green	round	7-10

Boldface-lettered: species for which foraging data were analyzed

* species not subjected to colorimetrical measurement and not included in Figure 4

† dehiscent and bright orange aril: monkeys mostly approached to non-dehiscent stage of the fruits

‡ dehiscent and bright red aril: monkeys mostly approached to non-dehiscent stage of the fruits

¶ reflectance spectrum not measured

§ width of round and discoid fruits, length of cylindrical and oval fruits
